# S100B concentration in colostrums of Burkinabe and Sicilian women

**DOI:** 10.1186/1743-7075-5-15

**Published:** 2008-05-22

**Authors:** Maria Musumeci, Pasqua Betta, Emanuela Magro, Teresa Isaia, Jacques Simpore, Domenico MM Romeo, Salvatore Musumeci

**Affiliations:** 1 Department of Hematology, Oncology and Molecular Medicine, Istituto Superiore di Sanità (ISS), Rome, Italy; 2 Department of Pediatrics, Neonatal Intensive Care Unit, University of Catania, Italy; 3 Department of Microbiology, Policlinic Laboratory, University of Catania, Italy; 4 Medical Center San Camille, Ouagadougou, Burkina Faso; 5 Department of Neurosciences and Mother and Child Sciences, University of Sassari, and Institute of Biomolecular Chemistry, CNR, Li Punti (SS), Italy

## Abstract

The aim of this study is to determine the S100B concentration in colostrums of 51 Burkinabe and 30 Sicilian women, still living in their countries, and in case of a difference to search for its explanations, considering also ethnic differences.

The concentration of S100B, in colostrums of the first three days from the delivery, was assessed with commercial immunoluminometric assay.

The production of colostrums was significantly higher in Burkinabe women, where the colostrums S100B levels in the first day of lactation showed to be at 24 h higher than those of Sicilian mothers (672.21 ± 256.67 ng/ml vs 309.36 ± 65.28 ng/ml) and progressively decreased reaching the values of Sicilian mothers in the second and third day (204.31 ± 63.25 ng/ml and 199.42 ± 45.28 ng/ml, respectively). Correlation was found between the level of S100B and the length of stage II (duration of expulsive phase of delivery), but the correlation with pain was found only in Burkinabe women.

The S100B level in colostrums of Burkinabe mothers differs from that of Sicilians only in the first day of lactation, and in consideration that Burkinabe women produce more colostrums, their newborns receive, during the first days of life, an higher amount of S100B. The elevated quantity of S100B ingested by Burkinabe newborn in the first days of life could promote the physiological postnatal brain adaptation and maturation in the precarious delivery condition of African infants.

## Background

Human milk contains a multitude of substances that guaranty the adaptation of newborns to the extra-uterine life, whose concentrations are regulated by a biological watch modulated by neuroendocrine and immune factors [1-3].

S100B is an acidic vitamin D-dependent calcium-binding protein, involved in the regulation of all aspect of cell function, largely present in the nervous system, being highly concentrated in glial cells [4,5]. Recently this protein has been isolated in different cells and biological fluids including the human milk [6]. Different function have been attributed to this protein, but the most interesting is that it acts as a cytokine [7] with a neurotrophic effect regulating the brain maturation in all the biochemical, morphological and electrophysiological expression [8]. Several reports underline the role of plasma S100B as a markers of neonatal asphyxia [9,10]. It could be the expression of white matter damage, involving for a more long time oligodendroglia differentiation, and the dosage of S100B became important in the monitoring and follow up of preterm with neurological damage [9].

The role of S100B in the relationship between mother and newborn is complex and continues after delivery through the secretion of this protein in breast milk. S100B is released in relatively large amounts into both the maternal and foetal circulation during labour and a correlation between S100B in cord blood levels and the physiological events related to brain maturation has been documented [10-12]. Recently Schulpis et al [13] investigated the effect of the mode of labour and delivery on the protein S100B serum concentrations in mothers and their newborns and demonstrated an increase of S100B serum levels in the mothers who delivered with prolonged labour for natural way. This increase was associated to long lasting, oxidative and/or psychogenic stress, while remarkably high levels of S100B in the newborns could be due to compressive conditions on the foetus brain during this mode of delivery [9].

After delivery, when the lactation starts, S100B is secreted in human milk at a level 80–100 times that in mother plasma, but the function of S100B content in colostrums is still unknown [14]. No data on the absorption of S100B in maternal milk by infants and on the effect of a potential contribution of exogenous S100B to measurements of the protein in human milk are reported

The S100B increases Ca^2+ ^absorption by buffering Ca^2+ ^in the cytoplasm and increases ATP-dependent Ca^2+ ^transport in duodenal membrane vesicles, involving second messengers, such as cAMP, cGMP, G-proteins, diacylglycerol through a phosphorylation cascade. This type of signalling involves covalent protein modification depending on two enzyme systems, protein kinases and phosphatases, and consequently, operates at a slower time scale than Ca^2+ ^signalling [15]. This role of S100B in the regulation of Ca^2+ ^transport through intestine epithelium is important in the postnatal adaptation of newborn as well as it was demonstrated by our group for the colostrum beta endorphin (EP) content [16]. In fact Beta-EP decreases the intracellular concentration of free Ca^2+ ^ions in brain [17], thus providing endogenous analgesia during labour in the mothers [18] and consequently in newborns.

Gazzolo et al [19], reported that S100B levels correlate with the gestational age and progressively increase during the maturation phases of lactation, differently from beta-endorphin which decrease rapidly during the first three days of lactation [19]. Since this peptide continues to maintain its maximum effect on neonates during all the period of lactation and the volume of milk ingested by infants increases during the lactation, it is conceivable that the S100B content in breast milk follow the brain maturation of breast feeding infants.

The aim of the current study was to determine the S100B concentration in human colostrums of Burkinabe and Sicilian mothers in the first three days post-partum and to correlate S100B levels with the anthropologic characteristics of mothers, the modality of delivery, the pain and the psychological involvement.

## Methods

### Study area

Colostrum samples from 51 Burkinabe women were collected between July and October 2005 at the Maternity Centre Medical Saint Camille (CMSC) in Ouagadougou (Burkina Faso – Africa), where 25–30 deliveries occur daily. Burkina Faso (formerly Upper Volta) was once a French colony, but it gained its independence in 1960 and is currently one of the poorest countries in the West Africa. The population of 11–12 million people belongs to several ethnic groups (Mossi, Peuhul, Gurunsi, Bobo, etc). They are primarily shepherds or non-nomadic farmers and live in sod and thatch huts of small and rural villages. Their socio-economic status is poor and their hygienic-sanitary conditions are defective with a bad water supply. Colostrum samples from 30 Sicilian women were collected between October and November 2005 at the Maternity of St Bambino Hospital of Catania, which is located in East Sicily, Italy.

All samples were from mothers who delivered at term vaginally. Ethical approval for the study was received by the institutional review board at the CMSC and St Bambino Hospital.

### Subjects

Data on anthropologic characteristics of mothers (age, number of deliveries, gestational age, child birth preparation, assistance to delivery, length of delivery periods, obstetric complications such as episiorraphy, infections, requirement of antibiotics, pain or psychological involvement) were collected from all the participants to the study. Informations on the socioeconomic status were also collected at the admission to the Maternity in Ouagadougou and Catania. The child birth preparation was made only by Sicilian mothers with the help of a midwife, who often was the same during delivery. Sicilian mothers received a single injection of an ergot derivative (0.2 mg), a powerful vasoconstrictor, immediately after delivery.

The exclusion criteria included HIV infection, sexual transmitted diseases and mastitis.

All individuals participating in the study signed informed consent forms.

No epidurals or other types of analgesia were given to mothers of either country.

The psychological involvement was quantified by a semi structured interview within the first 24 h from delivery:

- Low pain score (1): correspond to moderately mechanical low back pain, which was accepted by mothers as a natural consequence of delivery;

- High pain score (2): correspond to pain in all part of abdomen, which determined a suffering psychological status of the mothers.

Mother nutrition was in accord to the traditional habits of their countries.

### Milk sample collection

Mothers for colostrum donation were chosen in order of presentation at the Maternity Department. Table 1 summarizes the characteristics of breast milk sample donors. Samples were collected within the first 24 h post partum and at 24 h of interval up to the third day, by the same teams both in Italy and in Burkina Faso using a standardized procedure: before breastfeeding their babies, breast milk was collected for 10 minutes by hand squeezing in 2 ml fractions tubes, the sterile polystyrene tubes, when full, were refrigerated at 4° in polystyrol box containing ice and then immediately transferred to laboratory where they were stored at -80°C.

**Table 1:** Anthropometric, obstetrics and delivery characteristics, socio economic status of mothers

	Sicilians n. 30	Burkinabe n. 51	P-value
Maternal age (years)*	27.0 ± 6.8	26.6 ± 7.0	NS
Height (m)*	1.61 ± 0.06	1.62 ± 0.05	NS
BMI (kg/m^2^)*	22.3 ± 4.5	23.0 ± 4.0	NS
N. Delivery	2 (1/3)	4 (1/9)	0.0001
Child birth preparation	30	0	0.0001
Gestational Age (weeks)	40.0 (39–41)	39.5 (38–40)	NS
Vaginal Delivery	30	51	NS
Assistance to delivery	30	51	NS
Episiorraphy	30	0	0.0001
Lenght of stage II (min)	45(20–70) 45.3 ± 11.53	30(15–60) 29.66 ± 10.6	0.0001
Ergotamine injection	30	0	0.0001
Obstetrics Complication (Infection, antibiotic use)	0	0	NS
Homes (%) with			
No electricity	0	68.2	0.0001
No refrigerator or freezer	1	82.1	0.0001
No private water supply	0	73.2	0.0001
No private toilets	1	87.0	0.0001
Radio set	100	88.2	NS
TV set	99.0	26.7	0.0001

BMI – body mass index; NS – not significant.

Values given as mean ± standard deviation or median and range

This operation was repeated for three consecutive days at 24 h intervals.

Then, the samples were transferred in dry ice to the Laboratory of Policlinic, University of Catania, Italy. After thawing, colostrum samples were first centrifuged at 680 × g for 10 min at 4°C. The liquid component was removed and re-centrifuged at 10,000 × g for 30 min at 4°C. The floating lipid layer and cellular sediments were removed. After separation, the milk serum fraction of colostrums samples was stored in 1.5 ml Eppendorph tubes and frozen at -80°C to be used in our assays. The time from the collection of colostrums and the assays was less than 30 days.

### S100B assay

The S100B protein concentration was measured in all samples using a commercially available immunoluminometric assay (Liaison Sangtec 100 Diasorin S.p.A. Saluggia (VC)-Italy). According the manufacturer's indication this assay is specific for the beta subunit of protein known to be predominant (80–90%) in the human brain. The intra-assay variation coefficient (repetitively) was < 5%; the inter-assay variation coefficient (reproducibility) was < 10%. A standard curve was added to each plate; the reported results were the mean of two determinations.

### Statistical analysis

Colostrum S100B levels were presented as mean ± standard deviation. Statistical comparison of S100B concentrations among the samples collected over three consecutive days were performed using non parametric Wilcoxon rank test for paired and unpaired samples. The correlation between S100B and the length of stage II was made through linear regression curve and calculation of r.

The power of samples calculated at a significance limit < 0.05 with Statmate 2 program for Windows (GraphPad Prism ver 4, USA) was > 60%. A p-value < 0.05 was selected for significance in all the statistical tests.

## Results

### Anthropological characteristics

The characteristics of Burkinabe and Sicilian mothers are summarized in Table 1. Burkinabe mothers live in very precarious social and economical conditions (p < 0.0001) and had a greater number of deliveries (4 vs 2 p < 0.0001). The gestational age was comparable and the delivery was by natural way. Only Sicilian women received child birth preparation, but all Sicilian and Burkinabe women were assisted by a midwife during delivery. The labour duration of Burkinabe women was medially 7 (5–8) hours and that of stage II (duration of expulsive phase of delivery) 30 min (15–60 min), while those of Sicilian women was longer (6–9 hours, mean 8) and that of stage II was 45 min (20–70 min). Episiorraphy was performed routinely to Sicilian women, who received immediately post partum a ergot derivative injection.

### S100B determination

Results of S100B determinations (ng/ml) are reported in Table 2.

**Table 2:** S100B in the colostrum of Burkinabe and Sicilian women, expressed as ng/ml and volume in ml/10 min.

**Samples**	**Parameter**	**S100B (ng/ml) and volume (ml/10 min.)**		
		1° day	2° day	3° day

Burkinabe women n.51 (A)	S100B (ng/ml)	672.21 ± 256.67^*	204.31 ± 63.25	199.42 ± 45.28
	Volume (ml/10 min.)	6.0 ± 0.5^*	8.0 ± 0.5^**	10 ± 0.5^
Sicilian women n.30 (B)	S100B (ng/ml)	309.36 ± 65.28*	205.16 ± 39.15	190.25 ± 46.23
	Volume (ml/10 min.)	2.0 ± 0.1*	4.0 ± 0.1**	6.0 ± 0.1

A → B ^ P < 0.0001;*1° → 2° day P < 0.0001; ** 2° → 3° day P < 0.0001

Colostrum volumes collected from Burkinabe women were about 2–3 times larger than those from Sicilians (see Table 2).

The S100B mean concentration in the colostrums of Burkinabe women was 672.21 ± 256.67 ng/ml in the first day and progressively decreased in the second (204.31 ± 63.25 ng/ml) and third days (199.42 ± 45.28 ng/ml). S100B protein concentration in the colostrums of Sicilian women was lower (309.36 ± 65.28 ng/ml) than that observed in Burkinabe during the first day and decreased in the second (205.16 ± 39.15 ng/ml) and third day (190.25 ± 46.23 ng/ml) after delivery (Table 2).

### Correlation of S100B with modalities of delivery

No correlation was found between the level of S100B and the age of the mothers, gestational age, neither the number of pregnancy in both groups.

**Figure 1:**
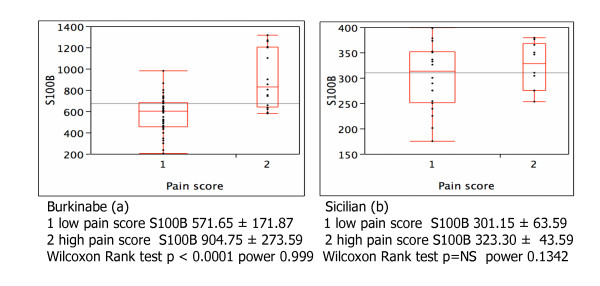
Correlation between colostrum S100B levels (ng/ml) in the first day of delivery and the pain score in Burkinabe (a) and Sicilian (b) women.

**Figure 2:**
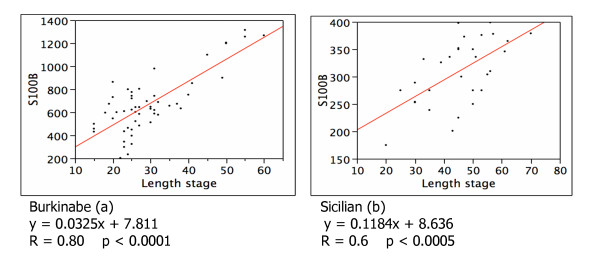
Correlation between colostrum S100B levels (ng/ml) in the first day of delivery and the lenght stage II in Burkinabe (a) and Sicilian (b) women.

The mean values of S100B (904.75 ± 273.59 ng/ml) were significantly higher in colostrums of 16 Burkinabe mother who showed a higher pain score (2) vs the mean values (571.65+171.87 ng/ml) of 35 mothers who showed a low pain score (1) (P < 0.0001) (see Figure 1a). The difference was not significant comparing Sicilian mothers with different pain score (301.15 ± 63.59 ng/ml in 20 mothers with low pain score and 323.3 ± 43.59 ng/ml in 10 mother with high pain score) (see Figure 1b). The correlation between S100B at the first day and the length stage II (see Figure 2a and 2b) was statistically significant in the two groups of Burkinabe and Sicilian mothers (P < 0,0001 and P < 0.0005 respectively).

Burkinabe women delivered without complications and came back home after three days. The Sicilian mothers did not show severe delivery complications, but all received episiorraphy.

## Discussion

The biological effects on newborns of elevated S100B content in human colostrums have not been fully elucidated [14]. We report the S100B levels in the colostrums of full term delivery Burkinabe and Sicilian women and our data show that S100B levels assessed in Burkinabe were significantly higher than those found in Sicilian colostrums only during the first day of lactation. Moreover our data show that the levels of S100B in the first three days are higher than that found by Gazzolo et al [14], in the colostrums of Caucasian women, collected an different time (starting 24 h after delivery). In such condition only after third day the values in ng/ml should be comparable between the two different studies. The differences which were observed with the values reported by Gazzolo et al [14] could be due to different modalities of execution, including thaw or freeze or to different kits. However the RLT/concentration curve with 10 points from 0.000 to 41.790 μg/L perfectly continuous confirms the reproducibility of this determination and the quality control samples included in the kits gave the expected values of 3.43 μg/L and 0.34 μg/L with a range of 2.74–4.12 and 0.24–0.44 respectively.

The socio economical factors do not seem influence the level of S100B in colostrums, which is maintained constant after the first day. In another study of our group the child birth preparation and the assistance to delivery by known midwives reducing the pain and the psychological stress decreased the Beta-EP-IRM contents in Sicilian colostrums when compared with Burkinabe colostrums [16]. Thus the elevated content of S100B in human colostrums could be important in overcoming birth stress, favouring the brain maturation [14], giving major protection to newborns in postpartum because of its neurotrophic role [20,21]. After this period the level of S100B decreased reaching similar concentration of Sicilians. On the contrary, S100B concentrations in colostrums of Sicilian women could be lower in the first day of lactation, probably due to the better assistance levels to mothers during delivery and to child birth preparation, with the consequent minor labour stress [13]. In fact the longer the delivery periods/the stronger the pain/the higher the stress, the higher would be the risk of neonatal tissue injury with the subsequent requirement of "stress adaptation", role that has been assigned to Beta-EP [16,22] and S100B [19] during the first days of lactation. However no correlation was found among Beta-EP-IRM and S100B both in Burkinabe and Sicilian colostrums, confirming that no relationship exist among these two proteins with regard to their regulation and gene expression.

The hypothesis that the abnormal elevation of S100B in colostrums of Burkinabe woman could be a consequence of immunological stress due to infections affected the mothers, was excluded by the fact that the mothers were admitted to this study only after exclusion of HIV infection, sexual transmitted diseases and mastitis. They show all a splendid health status.

The presence of S100B in breast milk could be in agreement with the characteristic of this protein which is known as a vitamin D-dependent calcium-binding protein (e.g alpha-lactoalbumin, calmodulin, osteocalcin) [23-25] in a biological fluid where the calcium is abundant and may be involved in the intestinal calcium absorption. For this reason the concentration of S100B is higher in breast milk compared to cord blood, peripheral blood, urine, cephalo rachidian fluid [12,26].

The progressive increment of S100B levels from colostrums to transition milk and mature milk demonstrates that this protein is important during the brain maturation of infants feed at breast, while in milk-formulae milks the S100B is at the lowest level reported in human milk [14], probably due to the protein epitopes modification during the production process. The reason of this difference is not known and may be object of future speculations.

This study demonstrates also that if the S100B levels are comparable in the colostrums of second and third day in Burkinabe and Sicilian mothers, Burkinabe women produce more colostrums (2–3 times) and so their newborns receive a higher S100B amount. This could promote the brain maturation in the precarious nutritional condition of Burkinabe mothers, whose colostrums contain lower levels of DHA 22:6n-3 (Docosahexaenoic acid) and LC n-3 PUFA (long-chain n-3 polyunsaturated fatty acids), a risk factor for future infant development ([27], in press).

In Sicilian mothers, the volume of drawn colostrums could be smaller because they received an Ergot derivative injection after delivery. An Ergot derivative is a dopamine receptor agonist which inhibits prolactin secretion, milk production and the initiation of breastfeeding by two to three days [28]. In our previous study prolactin levels present in colostrums of Sicilian and Burkinabe mothers remained stable in the following 3 days, while the content of IGF-1 in colostrums decreased progressively. These results underlie the essential role of prolactin at beginning of lactation [29], supporting the hypothesis of a racial difference in lacto genesis in these two sets of women.

Ethnic factors may impact the timing of lacto genesis stage II and it is possible that the Burkinabe mothers secreted milk sooner and in larger quantities than the European women [30], with the aim of major protection of their newborn.

These could be convincing explanations for volume difference and S100B content between Burkinabe and Sicilian colostrums in the first day of lactation.

## Conclusion

The S100B level in colostrums of Burkinabe mothers differs from that of Sicilians only in the first day of lactation, and in consideration that Burkinabe women produce more colostrums, their newborns receive, during the first days of life, an higher amount of S100B. The elevated quantity of S100B ingested by Burkinabe newborn in the first days of life could promove the physiological postnatal brain adaptation and maturation in the precarious delivery condition of African infants.

## Authors' contributions

MM and PB partecipated in the design and coordination of this study, EM and TI carried out the immunoluminometric assays, JS and SM collected the colostrum samples in Burkina Faso and in Sicily. DMMR performed the statistical analysis, SM conceived the study and wrote the manuscript.

All authors read and approved the final manuscript.
